# Optimizing External Beam Radiotherapy as per the Risk Group of Localized Prostate Cancer: A Nationwide Multi-Institutional Study (KROG 18-15)

**DOI:** 10.3390/cancers13112732

**Published:** 2021-05-31

**Authors:** Seo Hee Choi, Young Seok Kim, Jesang Yu, Taek-Keun Nam, Jae-Sung Kim, Bum-Sup Jang, Jin Ho Kim, Youngkyong Kim, Bae Kwon Jeong, Ah Ram Chang, Young-Hee Park, Sung Uk Lee, Kwan Ho Cho, Jin Hee Kim, Hunjung Kim, Youngmin Choi, Yeon Joo Kim, Dong Soo Lee, Young Ju Shin, Su Jung Shim, Won Park, Jaeho Cho

**Affiliations:** 1 Department of Radiation Oncology, Yongin Severance Hospital, Yonsei University College of Medicine, Yongin 16995, Korea; clickby_s@yuhs.ac; 2 Yonsei Cancer Center, Department of Radiation Oncology, Yonsei University College of Medicine, Seoul 03722, Korea; 3 Asan Medical Center, Department of Radiation Oncology, University of Ulsan College of Medicine, Seoul 05505, Korea; ysk@amc.seoul.kr (Y.S.K.); dfjnjoy@naver.com (J.Y.); kamea1004@naver.com (Y.J.K.); 4 Department of Radiation Oncology, Chonnam National University Hwasun Hospital, Chonnam National University College of Medicine, Gwangju 61469, Korea; tknam@chonnam.ac.kr; 5 Department of Radiation Oncology, Seoul National University Bundang Hospital, Seoul National University College of Medicine, Seongnam 13620, Korea; jskim@snubh.org (J.-S.K.); jbs.radonc@snubh.org (B.-S.J.); 6 Department of Radiation Oncology, Seoul National University Hospital, Seoul National University College of Medicine, Seoul 03080, Korea; jinhokim@snuh.org; 7 Department of Radiation Oncology, Kyung Hee University Hospital, Kyung Hee University College of Medicine, Seoul 02447, Korea; icarus070@hanmail.net; 8 Department of Radiation Oncology, Gyeongsang National University School of Medicine and Gyeongsang National University Hospital, Jinju 52727, Korea; blue129j@gnu.ac.kr; 9 Department of Radiation Oncology, Soonchunhyang University Seoul Hospital, Soonchunhyang University College of Medicine, Seoul 04401, Korea; changaram@schmc.ac.kr (A.R.C.); yh810530@hanmail.net (Y.-H.P.); 10 The Proton Therapy Center, National Cancer Center, Research Institute and Hospital, Goyang 10408, Korea; sulee126@ncc.re.kr (S.U.L.); kwancho@ncc.re.kr (K.H.C.); 11 Keimyung University Dongsan Medical Center, Department of Radiation Oncology, Keimyung University School of Medicine, Daegu 42601, Korea; jhkim@dsmc.or.kr; 12 Department of Radiation Oncology, Inha University Hospital, Inha University School of Medicine, Incheon 22332, Korea; cancerovercome@inha.ac.kr; 13 Department of Radiation Oncology, Dong-A University Hospital, Dong-A University School of Medicine, Busan 49201, Korea; cymin00@dau.ac.kr; 14 Department of Radiation Oncology, Kangwon National University Hospital, Chuncheon 24289, Korea; 15 Department of Radiation Oncology, College of Medicine, The Catholic University of Korea, Uijeongbu 11765, Korea; dreamdoc77@catholic.ac.kr; 16 Department of Radiation Oncology, Inje University Sanggye Paik Hospital, Seoul 04551, Korea; shinyj@paik.ac.kr; 17 Department of Radiation Oncology, Eulji Hospital, Eulji University School of Medicine, Seoul 01830, Korea; dcrystal@eulji.ac.kr; 18 Samsung Medical Center, Department of Radiation Oncology, Sungkyunkwan University School of Medicine, Seoul 06351, Korea

**Keywords:** prostate cancer, radiotherapy, NCCN, risk assessment, dose-escalation, hypofractionation

## Abstract

**Simple Summary:**

This multi-institutional study analyzed the patterns of care and outcomes of external beam radiotherapy (EBRT) in localized prostate cancer to identify the optimal EBRT strategy for each risk-stratified patient subgroup for clinical practice implementation. In 1573 patients from 17 institutions, EBRT treated prostate cancer effectively. Also, among various risk classification tools, NCCN classification revealed the highest predictive power. The modern RT techniques and dose escalation (≥179 Gy_1.5_) enhanced therapeutic effects of RT significantly, especially in the high-risk group. On the other hand, modest doses (≥170 Gy_1.5_) was a significant factor in the intermediate-risk group and no significant impact of dose was observed in the low-risk group. IMRT+ ≥179 Gy_1.5_+ hypofractionation resulted in higher biochemical failure-free survival in all risk groups, and it translated into survival benefits in the high-risk group. Therefore, risk-adapted RT (more intense RT, high-risk patients; moderate-dose RT, low-risk patients) can be considered, although further prospective studies are warranted.

**Abstract:**

Purpose: This nationwide multi-institutional study analyzed the patterns of care and outcomes of external beam radiotherapy (EBRT) in localized prostate cancer patients. We compared various risk classification tools and assessed the need for refinements in current radiotherapy (RT) schemes. Methods and Materials: We included non-metastatic prostate cancer patients treated with primary EBRT from 2001 to 2015 in this study. Data of 1573 patients from 17 institutions were analyzed and re-grouped using a risk stratification tool with the highest predictive power for biochemical failure-free survival (BCFFS). We evaluated BCFFS, overall survival (OS), and toxicity rates. Results: With a median follow-up of 75 months, 5- and 10-year BCFFS rates were 82% and 60%, and 5- and 10-year OS rates were 95% and 83%, respectively. NCCN risk classification revealed the highest predictive power (AUC = 0.556, 95% CI 0.524–0.588; *p* < 0.001). Gleason score, iPSA < 12 ng/mL, intensity-modulated RT (IMRT), and ≥179 Gy_1.5_ (EQD2, 77 Gy) were independently significant for BCFFS (all *p* < 0.05). IMRT and ≥179 Gy_1.5_ were significant factors in the high-risk group, whereas ≥170 Gy_1.5_ (EQD2, 72 Gy) was significant in the intermediate-risk group and no significant impact of dose was observed in the low-risk group. Both BCFFS and OS improved significantly when ≥179 Gy_1.5_ was delivered using IMRT and hypofractionation in the high-risk group without increasing toxicities. Conclusions: With NCCN risk classification, dose escalation with modern high-precision techniques might increase survivals in the high-risk group, but not in the low-risk group, although mature results of prospective studies are awaited.

## 1. Introduction

External-beam radiotherapy (EBRT) is a well-established local therapy for non-metastatic prostate cancer [1,2]. EBRT alone, or with other treatments, is highly effective for prolonging life and preserving quality of life. Localized prostate cancer patients can be stratified into risk groups by clinicopathological parameters. Each risk group has multiple treatment options; the superiority of different treatments remains undetermined. Since the publication of the D’Amico risk-stratification system [3], several risk-stratification tools have been proposed to facilitate treatment decisions for prostate cancer [4,5,6,7].

Traditionally, prostate cancer patients receive a 64–70 Gy EBRT dose, although no clear cutoff is recommended. Recent studies suggested these doses to be insufficient for tumor control, and dose escalation was proposed for the best biochemical control with the development of modern RT techniques [8,9,10,11,12,13,14,15,16,17,18,19]. Reports indicate that intensity-modulated radiotherapy (IMRT), which has largely replaced non-modulated three-dimensional conformal radiation therapy (3D-CRT), delivered higher radiation doses with reduced toxicity, without compromising treatment outcomes [20,21,22]. Since prostate cancer has a low *α*/*β* ratio (1.5) [23], treating patients with higher doses/fraction over shorter periods (hypofractionation (HF)) using IMRT has generated increased interest [24]. Nevertheless, the optimum dose and fractionation remain unclear. Moreover, studies analyzing the effects of dose escalation with modern RT techniques in each risk group are scarce.

Our study was designed to comprehensively evaluate these gaps in the literature using a nationwide database of localized prostate cancer patients. We first compared various risk classification tools in our cohort. Ultimately, we aimed to identify the optimal EBRT strategy for each risk-stratified patient subgroup and to identify risk-adapted treatment policies for clinical practice implementation.

## 2. Materials and Methods

### 2.1. Patient Selection

Patients with histologically confirmed non-metastatic prostate cancer who underwent definitive EBRT in Korea (January 2001 to December 2015) were eligible for this study. Patients with <3 years’ follow-up, insufficient EBRT data, salvage RT for any recurrence, or previous pelvic irradiation, prostate brachytherapy, or cytotoxic chemotherapy were excluded. The database included 1573 patients’ data from 17 different institutions. We reviewed medical charts and analyzed disease status, treatment, and outcomes.

All patients were re-grouped into risk subgroups per the following risk-stratification tools: National Comprehensive Cancer Network (NCCN) [4], D’Amico [3], American Urological Association (AUA) [5], and Cambridge Prognostic Groups (CPG) risk group systems [6]. Table S1 describes these tools. We selected the tool that exhibited the highest predictive power for our data and classified all patients into low-, intermediate-, and high-risk groups according to the risk classification.

The Korean Radiation Oncology Group (KROG) authorized and cooperated with this study (named “KROG 18-15”). The institutional review boards of each participating hospital approved KROG 18-15. Since the study was retrospective, the need for written informed consent was waived.

### 2.2. Treatment and Follow-Up

All patients underwent EBRT. The techniques and doses were determined by the attending radiation oncologists. RT techniques included 3D-CRT, IMRT, and proton beam therapy. The dose fractionation scheme included conventional fractionation (CF) (1.8–2 Gy/fraction), moderate-HF (>2 Gy/fraction), and ultra-HF (≥5 Gy/fraction). To adjust for different dose fractionation, we calculated the biologically effective dose (BED) with prostate cancer *α*/*β* ratio of 1.5. Concerning the RT field, most patients received radiation to the prostate (±seminal vesicle); the whole pelvis was added for some patients with adverse features. Androgen deprivation therapy (ADT) was administered with RT or before the referral for RT. Treatment guidelines for each risk group regulated the ADT protocol. Briefly, in patients in the unfavorable intermediate-risk, high-risk, and very-high-risk groups, hormone therapy was administered first, unless there were contraindications. The duration of ADT was usually 2 to 6 months in the low/intermediate-risk group. In some patients, 2 months of neoadjuvant ADT was performed before the start of RT. The duration was longer (usually 2 to 3 years) in the high/very-high-risk group. Also, neoadjuvant ADT (within 6 months) was performed in some patients. However, ADT duration was usually determined according to changes in PSA levels, and there were differences in each institution’s policies. The attending physician (mainly, urologists) of each institution determined ADT maintenance duration and regimen.

Per institutional protocols, patients were followed up after RT completion at 4–6 weeks, 3–6 monthly until the end of year 2, and 6–12 monthly thereafter. Prostate-specific antigen (PSA) measurement was performed at every follow-up. Bone scintigraphy and/or computerized tomography scans were performed to identify distant metastases including lymph node metastasis, if clinically indicated. PSA relapse was defined using the Phoenix definition (nadir + 2 ng/mL). ADT was reinitiated if PSA was persistently elevated after RT in some patients even though PSA was not nadir + 2 ng/mL. Therefore, biochemical failure (BCF) was defined as nadir + 2 ng/mL or ADT initiation for persistently elevated PSA. Clinical failure included any type of disease progression diagnosed by radiological/histological examinations. Radiation-related lower gastrointestinal (GI) or genitourinary (GU) toxicity was assessed during RT, the first month after RT, and subsequently at 3- to 6-month intervals (Radiation Therapy Oncology Group radiation toxicity criteria). Events occurring within and 3 months post-RT were classified as acute and late toxicities, respectively.

### 2.3. Statistical Analysis

The primary endpoint was BCF-free survival (BCFFS): the interval between the date of diagnosis and the date of BCF or last follow-up. The secondary endpoints were the overall survival (OS) and cancer-specific survival rates. The OS rate was defined as the time from the date of diagnosis to death due to any cause or the last follow-up. The cancer-specific survival rate only accounted for deaths due to prostate cancer. Survival rates were estimated using the Kaplan–Meier method. To identify the highest predictive power among the risk-grouping systems, we generated receiver operating characteristic curves for BCFFS in each system and estimated the area under the curve (AUC). The prognostic impact of clinical or RT-related factors was analyzed (log-rank test, categorical variables; logistic regression analysis, continuous variables) in all patients or in each risk subgroup. All variables that showed statistical significance in univariate analyses were entered into multivariate analyses using a Cox proportional hazard model. The most significant radiation dose (Gy) cutoff was determined by categorizing prescribed doses per patient into two groups using the cutoff point with the highest Youden’s index value, and the patients were divided into “high-dose” and “low-dose” groups. Other subgroup characteristics were compared using Pearson χ2 test, Fisher exact test, and Student’s *t*-test. *p*-values < 0.05 denoted statistical significance. Furthermore, we used propensity score matching to estimate the average marginal effect of RT modality (3D vs. IMRT/proton) on those who received it, accounting for confounding by the included covariates. We performed 1:1 nearest-neighbor propensity score matching without replacement with a propensity score estimated using logistic regression of the treatment on the covariates. After matching, we confirmed that all standardized mean differences for the covariates were below 0.1 and that all standardized mean differences for squares and two-way interactions between covariates were below 0.15, indicating adequate balance. The propensity score was estimated using logistic regression of the RT modality (3D vs. IMRT/proton) on the covariates. We conducted propensity score matching using the Matchlt package in R [25]. All other analyses were performed using SPSS version 25.0 (IBM Inc., Armonk, NY, USA).

## 3. Results

### 3.1. Patient Characteristics

From 2001 to 2015, we enrolled 1573 patients per the inclusion criteria. Each risk group had the following number of patients: (1) NCCN: low, 177; intermediate, 408; high, 761; very high, 227. (2) D’Amico: low, 177; intermediate, 383; high, 1013. (3) AUA: low, 177; intermediate, 539; high, 857. (4) CPG: low, 178; intermediate, 407; high, 366; very high, 622. In total, 63% received ADT before RT and/or concurrently. Concurrent ADT was performed in 61% NCCN high-risk group patients and 55% NCCN very-high-risk group patients. Whole pelvis RT (WPRT) was performed in 34% of patients, predominantly in high-risk patients (NCCN high risk 47%; NCCN very high risk, 53%). With regards to RT modality, 3D-CRT, IMRT, and proton therapy were performed in 24%, 74%, and 3% of patients, respectively. With regards to RT dose scheme, conventional fractionation, HF, and ultra-HF were selected for 44%, 47%, and 9% of patients, respectively. The median total irradiated dose was 179.1 (range, 107.3–225.0) Gy_1.5_. Significantly higher doses were delivered with the selection of more hypofractionated schedules (D_mean_: conventional, 167.9 Gy; HF, 185.5 Gy; and ultra-HF, 214.9 Gy; *p* < 0.001) (Figure S1a) or more modern RT techniques (D_mean_: 3D, 166.0 Gy; IMRT, 185.1 Gy, and proton therapy, 191.3 Gy; *p* < 0.001) (Figure S1b). The use of modern RT techniques (IMRT or proton therapy, HF or ultra-HF, and higher dose) had increased in more recent years (Figure S2). Table 1 summarizes patient characteristics.

### 3.2. Treatment Outcomes and Risk Stratification

**Figure 1:**
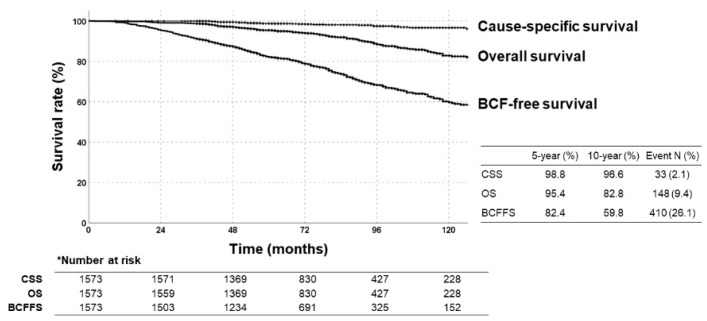
Kaplan–Meier survival curves of biochemical failure (BCF)-free survival, overall survival (OS), and cause-specific survival rates in all patients.

The median follow-up was 75 (36–239) months from the first diagnosis. The 5- and 10-year rates were as follows (Figure 1): BCFFS; 82.4%, 59.8%, respectively. OS; 95.4%, 82.8%, respectively. Cause-specific survival rates; 98.8%, 96.6%, respectively. Of 148 deaths, most were age-related, 33 were disease-related. At a median of 46 (2–211) months after the first diagnosis, 19.3% of patients developed BCF events, with/without clinical recurrence. Table S2 shows details of recurrence patterns.

**Figure 2:**
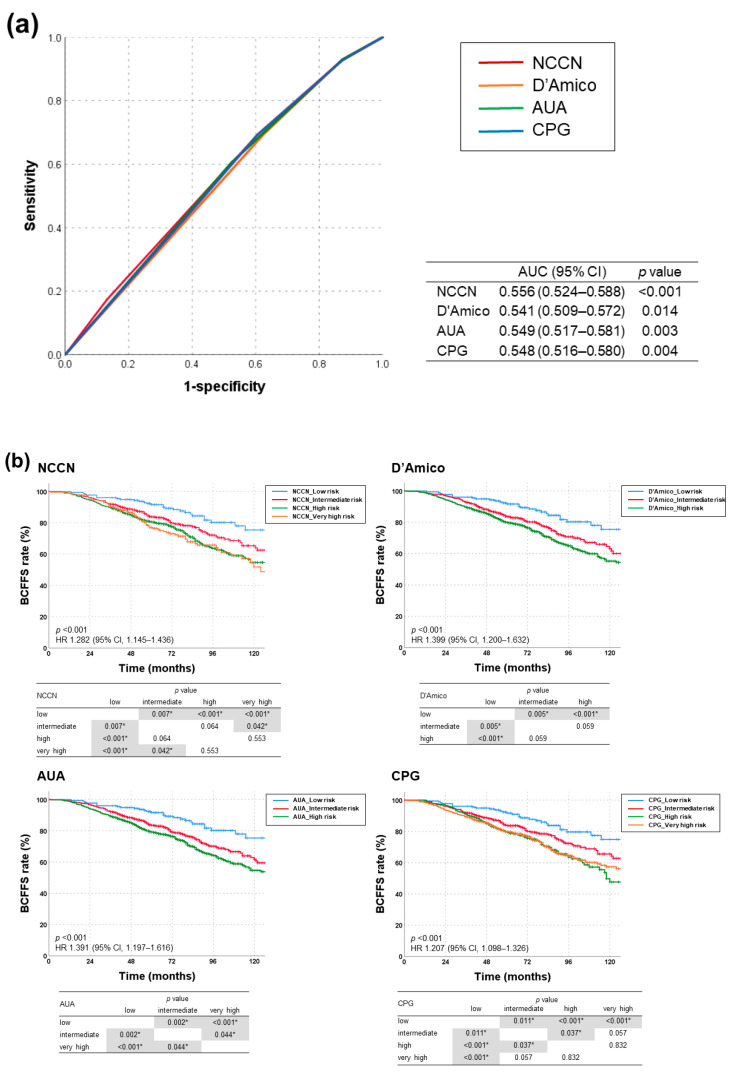

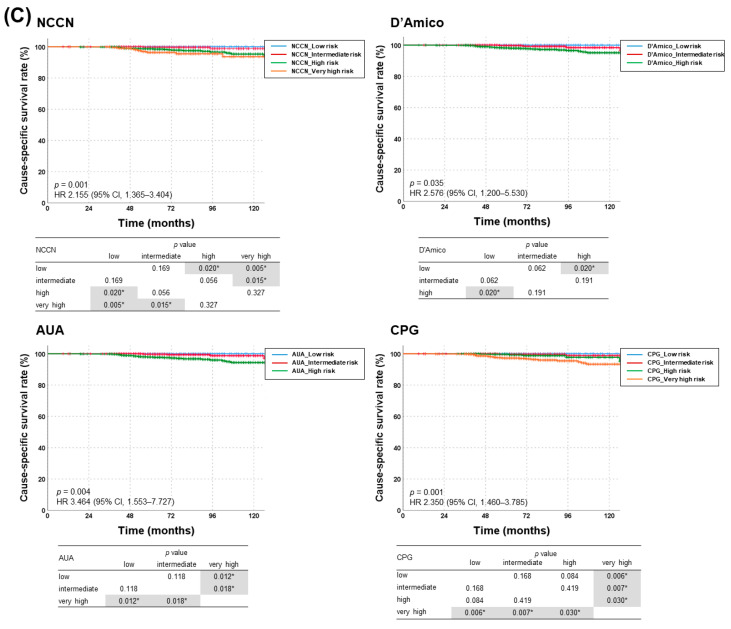
(**a**) Receiver operating characteristic curves for each risk-stratification tool as a predictor of biochemical failure (BCF)-free survival, and (**b**) BCF-free survival and (**c**) cause-specific survival rates according to risk groups stratified using four different classification tools (NCCN, D’Amico, AUA, and CPG guidelines). AUA, American Urological Association; CPG, Cambridge Prognostic Groups; NCCN, National Comprehensive Cancer Network. * denotes statistically significant *p* value.

AUC values for BCFFS rates were highest with NCCN risk classification (0.556, 95% confidence interval (CI): 0.524–0.588; *p* < 0.001), followed by AUA, CPG, and D’Amico (Figure 2a). All risk-stratification tools differentiated BCFFS rates (Figure 2b) and cause-specific survival rates (Figure 2c) significantly. OS rates were not significantly different in all risk groups (NCCN: *p* = 0.387, hazard ratio (HR) = 1.085, 95% CI = 0.902–1.305; D’Amico: *p* = 0.576, HR = 1.121, 95% CI = 0.885–1.420; AUA: *p* = 0.589, HR = 1.097, 95% CI = 0.868–1.386; CPG: *p* = 0.712, HR = 1.070, 95% CI = 0.918–1.246). Appendix A summarizes the BCFFS, BCF, OS, and cause-specific survival rates in each NCCN risk group.

### 3.3. Prognostic Factors in Each NCCN Risk Group

Univariate and multivariate analyses for BCFFS in all patients revealed that Gleason score, initial PSA <12 ng/mL, RT modality (IMRT/proton), and RT dose ≥179 Gy_1.5_ were independent significant factors (*p*; 0.016, <0.001, 0.036, 0.022, respectively) (Table 2). BED and BCFFS showed a significant linear relationship (*p* = 0.026), with 179 Gy_1.5_ (equivalent dose in 2 Gy fractions (EQD2), 77 Gy)) as the most significant cutoff. After propensity score matching according to RT technique (3D vs. IMRT/proton), no RT-related factors were significant for BCFFS. Only Gleason score, initial PSA, and ADT were independent significant factors (*p* < 0.001, 0.003, <0.001, respectively). Patient characteristics of the matched groups are shown in Table S3, and the results of the analyses are shown in Table S4.

NCCN low-risk group: No significant prognostic factor for BCFFS or OS was identified, and no significant impact of a higher dose was observed. NCCN intermediate-risk group: ADT combination, HF/ultra-HF, and ≥170 Gy1.5 (EQD2, 72 Gy) were significant favorable factors for BCFFS in univariate analysis (*p*; 0.047, 0.013, 0.014, respectively); only ADT was significant in multivariate analysis (*p* = 0.028, HR = 0.606, 95% CI = 0.388–0.947). On the other hand, there was no significant factor for OS in both univariate and multivariate analyses. NCCN high-risk group: ADT (*p* < 0.001, HR = 0.505, 95% CI = 0.379–0.672), IMRT/proton therapy (*p* = 0.014, HR = 0.638, 95% CI = 0.446–0.915), and ≥179 Gy1.5 (*p* = 0.009, HR = 0.503, 95% CI = 0.300–0.844) were independent significant factors for BCFFS in both univariate and multivariate analyses. For OS, although IMRT/proton therapy (*p* < 0.001, HR = 0.367, 95% CI = 0.226–0.596), CF (*p* = 0.002, HR = 2.271, 95% CI = 1.353–3.811), and ≥179 Gy1.5 (*p* = 0.003, HR = 0.466, 95% CI = 0.282–0.770) were significant factors in univariate analysis, none was significant in multivariate analysis. NCCN very-high-risk group: Gleason score and HF/ultra-HF were significant factors for BCFFS in univariate analysis (*p* = 0.019, 0.038, respectively), but not in multivariate analysis. Similarly, no factor was significant in univariate and multivariate analyses for OS. Similar results were shown even after propensity score matching (Table S4).

### 3.4. Radiotherapy-Related Factors

**Figure 3:**
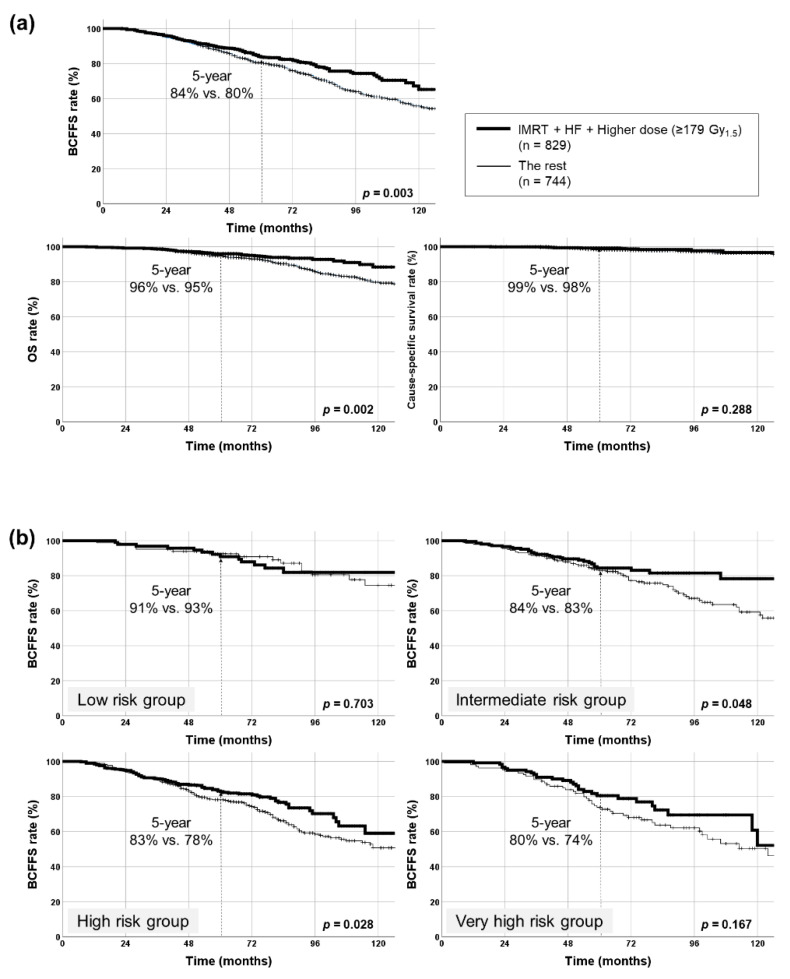

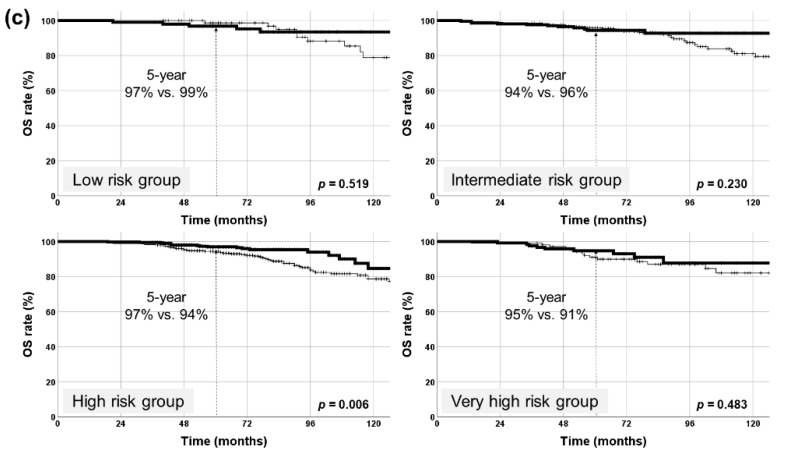
Kaplan–Meier survival curves of patients treated with radiotherapy using intensity-modulated radiotherapy (IMRT), hypofractionation (HF), and higher dose (≥179 Gy_1.5_). (**a**) Biochemical failure (BCF)-free survival (BCFFS), overall survival (OS), and cause-specific survival rates in all patients (829 patients with all three factors vs. the rest). BCFFS and OS rates were significantly improved with this combination, but no significant differences were observed in cause-specific survival rates. (**b**) BCFFS rates in each NCCN risk group. BCFFS rates were significantly improved with this combination in intermediate- and high-risk groups. (**c**) OS rates in each NCCN risk group. OS rate was significantly improved with this combination only in the high-risk group. NCCN, National Comprehensive Cancer Network. * denotes statistically significant p value.

RT comprising IMRT, HF, and higher dose (≥179 Gy_1.5_) was administered to 829 (53%) patients. BCFFS and OS rates significantly improved in these patients (*p* = 0.003, 0.002, respectively) (Figure 3a). Cause-specific survival rates did not have significant differences (*p* = 0.288). Following patient subgrouping (NCCN classification), this triple combination (IMRT + HF + ≥179 Gy1.5) elicited a significant impact on BCFFS in the NCCN intermediate- and high-risk groups (*p* = 0.048, 0.028, respectively). HR for BCFFS was largest in the NCCN high-risk group (Figure 3b, Table 3). Also, this combination decreased BCF rates significantly in NCCN intermediate-, high-, and very-high-risk groups (*p* = 0.004, 0.010, 0.007, respectively): the HR for BCF rate was largest in the NCCN high-risk group (Table 3). This combination significantly increased OS rate only in the NCCN high-risk group (*p* = 0.006, HR = 0.477, 95% CI = 0.279–0.815) (Figure 3c).

### 3.5. Toxicities

Radiation-related toxicity (≥grade II) rates were acceptable (acute GU, 10.7%; acute GI, 6.2%; late GU, 13.4%; late GI, 6.5%). There was no significant increase in toxicity in patients with a longer follow-up duration (Table S5). Multivariate analysis revealed that acute GU toxicity (≥grade II) incidences decreased significantly with ultra-HF (CF, 10.1%; HF, 13%; ultra-HF, 1%; *p* = 0.006) and IMRT (10% vs. 13%, *p* = 0.054). Acute GI toxicity (≥grade II) incidences increased significantly with WPRT (11% vs. 4%, *p* < 0.001). Late GU toxicity (≥grade II) incidences were significantly higher with HF than with CF (19% vs. 8%, *p* = 0.035) and with WPRT than with prostate-only RT (22% vs. 9%, *p* < 0.001). IMRT/HF/dose escalation did not increase grade III toxicities.

IMRT + HF + higher dose (≥179 Gy_1.5_) had the following toxicity (≥grade II) rates: acute GU, 15.3%; acute GI, 7.9%; late GU, 22.7%; late GI, 6.3%. The late GU toxicity rate was higher than that in all patients. Most events were grade II, and the grade III toxicity rates were similar (Table S6).

## 4. Discussion

Our study showed a significant linear relationship between total radiation dose and BCFFS with 179 Gy_1.5_ (EQD2, 77 Gy) as the most significant cutoff. Dose escalation prominently benefited the high-risk group. In the intermediate-risk group, modest doses over 170 Gy_1.5_ (EQD2, 72 Gy) delivered significantly better outcomes. IMRT + dose escalation + HF resulted in 5-year BCFFS rates of 80–90% in all risk groups; the improvement in BCFFS translated into survival benefits in the high-risk group. Although HF increased the late GU toxicity rate, IMRT/HF/dose escalation did not increase grade III toxicities. The combination of contemporary RT techniques did not increase acute/late toxicities significantly.

This study holds both similarities and differences from existing studies. We predominantly analyzed RT-related factors in each risk group to derive conclusions for application in clinical practice. Before analyses, patients were classified into low-, intermediate-, and high-risk groups (NCCN classification). Consistent with previous reports, we confirmed that dose escalation benefited BCF, most prominently in the high-risk group. Higher RT dose did not increase ≥grade 2 RT-related toxicities. However, given the risk of toxicities, our data suggested using only moderate doses for low/intermediate-risk groups. Dose escalation was a significant factor for OS in the high-risk group, but not in multivariate analysis. However, together with IMRT and HF, dose escalation may be more effective, even at improving survival. It should not be forgotten that modern RT techniques have prognostic meaning when enabling dose escalation. Ultimately, the use of contemporary techniques may increase survival gains with intensified and risk-adapted RT.

Standard options for the initial management for localized prostate cancer include RT (EBRT and/or brachytherapy with or without ADT), radical prostatectomy, or active surveillance in select patient populations. In clinical practice, the choice of treatment is determined by various factors, including risk stratification, patient preference, clinicians’ judgment, and resource availability [1,2]. Based on NCCN guidelines [4], EBRT is a definitive initial treatment option in low-, intermediate-, and high-risk patients. EBRT may be considered post-surgery if there is a high risk of recurrence based on surgical pathology, increase in PSA levels during follow-up, or detection of local recurrence. Although there are no randomized trials comparing RT with radical prostatectomy yet, trials completed to date and observational data suggest that outcomes with either EBRT or brachytherapy are similar to those with radical prostatectomy when men with clinically localized prostate cancer are stratified based on clinical tumor (T) stage, pretreatment PSA, and Gleason score.

Various risk-stratification tools exist for selecting treatments for prostate cancer, and dividing patients into subgroups according to major risk factors to predict BCFs is recommended, and similarities between tools are evident [3,4,5,6,7]. The superiority of each tool for predicting prostate cancer-related deaths is unclear. In a recently published study, which used the Prostate Cancer database in Sweden [26], a population-based research database including both untreated and treated patients followed for prostate cancer-related deaths for up to 19 years, a total of nine pretreatment risk stratification tools were compared. The MSKCC nomogram (C-index: 0.81, 95% CI = 0.80–0.81), CAPRA score (C-index: 0.80, 95% CI = 0.79–0.81), and CPG system (C-index: 0.78, 95% CI = 0.78–0.79) exhibited the best performance for discriminating prostate cancer-related deaths. However, complete information on all variables used in the risk stratification tools were only available for 35% of the cohort. Furthermore, information on cT2 to T3 substages was not recorded in the database. In our nationwide database, we demonstrated that the NCCN system exhibited the highest AUC value for BCFFS, although the other classification systems demonstrated comparable significance. Future well-controlled studies with a larger sample size and long-term follow-up are warranted to identify the superiority of the different tools.

Various fractionation and dose regimens can be considered for RT depending on clinical conditions. The irradiated dose for definitive RT in localized prostate cancer was 70 Gy or lower in the past, but there have been attempts to improve treatment outcomes by increasing the radiation dose. Retrospective studies [8,9,10,11] reported increased BCFFS and OS rates with dose escalations. Randomized trials [10,13,14,15,16,17,18,19] reported significantly lower biochemical/clinical failure rates in the higher-dose arms than in the lower-dose arms. Nevertheless, these studies revealed greater late GI/GU toxicity rates with higher RT dose arm than in the lower-dose arm. Furthermore, although dose escalation significantly reduced BCFs, no improvements in OS were noted. A major limitation is that the therapeutic ratio of tumor control versus previously reported toxicity is less applicable at present given the employment of more modern treatment methods.

Multiple randomized trials and a meta-analysis evaluated the role of hypofractionated RT and concluded that its efficacy is equivalent to that of CF [27,28,29,30,31,32,33,34,35]. Data on whether HF increases overall treatment-related toxicity are inconclusive. Most trials [28,29,30,33] reported a small increase in acute GI toxicity risk. Two studies [34,35] identified a notable increase in late toxicity risk. A 2019 Cochrane review of 10 randomized trials concluded that the effects of HF on late GI toxicity were unclear and that there was little to no difference in acute/late GU toxicity [31]. Although the optimal regimen for HF remains unestablished, the AUA/ASTRO/ASCO guidelines [24] endorsed either 60 Gy/20 fractions or 70 Gy/28 fractions; most evidence supported these regimens. In our study, the most frequently selected schedule was 2.1–2.2 Gy/fraction or 2.5–2.6 Gy/fraction, although there were substantial differences between institutions. The total dose increased with an increase in dose/fraction, indicating that HF is useful for administering higher doses. Subsequent studies will help determine the optimal schedule to deliver higher RT doses.

Ultra-HF, also called stereotactic body radiation therapy (SBRT), is an appropriate alternative to CF RT in low/intermediate-risk prostate cancer patients who do not require nodal irradiation. Several prospective trials reported favorable efficacy and toxicities of ultra-HF [36,37,38,39]. Nevertheless, median follow-up time has generally been limited to 3–5 years, and there is concern regarding long-term outcomes and higher late GU toxicity with SBRT vs. IMRT. SBRT has not been directly compared with moderate HF. We were unable to demonstrate the benefits of HF over CF in BCFFS or BCF rates using multivariate analyses of the entire cohort. The significance of HF was larger in the intermediate- and high-risk groups than in the others. Therefore, radiation dose is a more important prognostic factor than fractionation schedule. Like previous reports, ultra-HF and HF increased acute and late GU toxicities (≥grade II), respectively. Thus, long-term follow-up and careful patient monitoring are necessary.

Our study has several limitations. First, no significant difference was identified in prognosis between the favorable and unfavorable intermediate-risk groups (data not shown). A potential reason was that “<50% positive cores,” the diagnostic criteria for the favorable intermediate-risk group, could not be identified in our retrospective database. As the classification of favorable and unfavorable groups was developed recently, precise classification of patients diagnosed in the past was difficult. Second, the omission or duration of ADT may not have reflected the modern standard of care for unfavorable intermediate- and high-risk patients. Further, salvage therapy was initiated at the clinician’s discretion, distinct to a standardized threshold. However, since we aimed to propose an optimal RT strategy, ADT-related analyses were not performed in detail. Third, it is necessary to demonstrate survival benefits or changes in long-term side effects via long-term follow-up, because disease-related events may occur even 5 and 10 years after diagnosis in prostate cancer patients. Therefore, a longer follow-up is essential to obtain solid conclusions with sufficient evidence to change clinical practice.

## 5. Conclusions

EBRT treats localized prostate cancer effectively. Also, the prognostic utility of NCCN risk grouping was validated in our nationwide cohort study. HF and IMRT may effectively deliver higher doses without significantly increasing severe toxicities. In the high-risk group, radiation dose escalation with modern high-precision RT techniques effectively increased survival rates, but not in the low-risk group. Risk-adapted RT (more intense RT, high-risk patients; moderate-dose RT, low-risk patients) can be considered, although further prospective, long-term follow-up studies are warranted.

## Figures and Tables

**Table 1:** Clinical and treatment characteristics of all patients (*n* = 1573).

Characteristics		No.	%
Age (year)		Median 73 (30–90)	
	≤70	592	39.9
	>70	981	60.1
Treatment year	2005	53	3.4
	2006	101	6.4
	2007	143	9.1
	2008	125	7.9
	2009	95	6.0
	2010	132	8.4
	2011	161	10.2
	2012	200	12.7
	2013	205	13.0
	2014	199	12.7
	2015	155	9.9
	2016	4	0.3
T stage	T1	216	12.1
	T2	680	39.6
	T3	618	42.7
	T4	56	5.4
	Tx	3	0.3
Gleason score		Median 7 (2–10)	
	≤6	464	29.6
	7	515	32.7
	8	346	22.0
	≥9	241	15.3
	Unknown	7	0.4
Initial PSA (ng/mL)		Median 26.3 (0.03–535.0)	
	<10	674	43.1
	10~20	366	23.4
	>20	525	33.5
NCCN risk group	Low	177	9.8
	Intermediate	408	22.9
	High	761	43.1
	Very high	227	12.9
D’Amico risk group	Low	177	11.3
	Intermediate	383	24.3
	High	1013	64.4
AUA risk group	Low	177	11.3
	Intermediate	539	34.3
	High	857	54.5
CPG risk group	Low	178	11.3
	Intermediate	407	25.9
	High	366	23.3
	Very high	622	39.5
ADT	No	543	34.5
	Before RT	210	13.4
	Before/concurrent RT	747	47.5
	Concurrent RT	27	1.7
	Post-RT	46	2.9
RT volume	Prostate (±SV)	1020	64.8
	Whole pelvis	534	33.9
	Half pelvis	8	0.5
	Unknown	11	0.7
RT modality	3D CRT	378	24.0
	IMRT	1119	71.1
	Proton therapy	44	2.8
	3D + IMRT	21	1.3
	Unknown	11	0.7
RT dose scheme	CF	692	44.0
	HF	738	46.9
	Ultra-HF	143	9.1
Fractional dose (Gy)		Median 2.2 (1.7–7.5)	
Total dose (Gy)		Median 72.6 (35.0–81.0)	
Total dose in BED (Gy_1.5_)		Median 179.1 (107.3–225.0)	

PSA, Prostate-Specific Antigen; NCCN, National Comprehensive Cancer Network; AUA, American Urological Association; CPG, Cambridge Prognostic Group; ADT, Androgen deprivation therapy; RT, Radiotherapy; SV, Seminal vesicle; 3D CRT, 3-dimensional conformal radiotherapy; IMRT, Intensity-modulated radiotherapy; CF, conventional fractionation; HF, hypofractionation; BED, Biologically effective dose; conventional fractionation: 1.8–2 Gy per fraction, moderate hypofractionation: >2 Gy per fraction, ultra-hypofractionation: ≥5 Gy per fraction.

**Table 2:** Results of univariate and multivariate analyses for biochemical failure-free survival in all patients.

Variable	Univariate Analysis		Multivariate Analysis	
	HR (95% CI)	*p* Value	HR (95% CI)	*p* Value
Age (continuous)	0.995 (0.981–1.008)	0.430		
Age (>70 vs. ≤70)	0.966 (0.793–1.177)	0.730		
Age (>60 vs. ≤60)	0.897 (0.634–1.269)	0.539		
T stage	1.142 (1.015–1.285)	0.027		
T3 vs. T1	1.434 (1.048–1.961)	0.024		
T2, T3, T4 vs. T1	1.389 (1.035–1.864)	0.029	0.817 (0.601–1.111)	0.198
Gleason score	1.160 (1.071–1.256)	<0.001		
≥9 vs. <9	1.501 (1.175–1.916)	0.003		
≥9 vs. <6	1.783 (1.332–2.386)	<0.001	1.359 (1.058–1.746)	0.016
≥9 vs. 7–8	1.784 (1.333–2.388)	<0.001		
7–8 vs. <6	1.303 (1.033–1.644)	0.025		
initial PSA	1.004 (1.002–1.006)	<0.001		
≥12 vs. <12	1.637 (1.343–1.996)	<0.001	1.508 (1.227–1.854)	<0.001
ADT combination (Yes vs. No)	0.744 (0.612–0.905)	0.003		
RT volume				
Prostate ± SV vs. Pelvis	0.970 (0.866–1.087)	0.604		
RT modality				
IMRT/Proton vs. 3D	0.719 (0.586–0.881)	0.001	0.761 (0.589–0.983)	0.036
Proton vs. 3D/IMRT	1.036 (0.644–1.666)	0.885		
RT dose scheme				
CF vs. HF/ultra-HF	1.346 (1.104–1.641)	0.003	0.773 (0.512–1.166)	0.220
HF vs. CF	0.723 (0.585–0.893)	0.003		
Ultra-HF vs. CF	0.840 (0.585–1.205)	0.343		
HF vs. ultra-HF	0.861 (0.592–1.252)	0.433		
Total dose, BED (Gy_1.5_) (continuous)	0.993 (0.988–0.999)	0.026		
BED ≥ 179 Gy_1.5_ vs. < 179 Gy_1.5_	0.701 (0.575–0.853)	<0.001	0.644 (0.441–0.939)	0.022

HR, Hazard ratio; CI, Confidence Interval; ADT, Androgen deprivation therapy; RT, Radiotherapy; SV, Seminal vesicle; 3D CRT, 3-dimensional conformal radiotherapy; IMRT, Intensity-modulated radiotherapy; CF, conventional fractionation; HF, hypofractionation; BED, Biologically effective dose; conventional fractionation: 1.8–2 Gy per fraction, moderate hypofractionation: >2 Gy per fraction, ultra-hypofractionation: ≥5 Gy per fraction.

**Table 3:** Results of Cox regression analysis for BCF-free survival and logistic regression analysis for BCF rate of combined radiotherapy-related factors (intensity-modulated radiotherapy, hypofractionation, and higher dose (≥179 Gy_1.5_)).

Subgroup	HR for BCFFS	95% CI	*p* Value
All patients	0.735	0.601–0.900	0.003
NCCN low-risk group	1.159	0.543–2.471	0.703
NCCN intermediate-risk group	0.650	0.423–1.000	0.048
NCCN high-risk group	0.731	0.553–0.966	0.028
NCCN very-high-risk group	0.712	0.441–1.152	0.167
	**HR for BCF Rate**	**95% CI**	***p* Value**
All patients	0.580	0.450–0.747	<0.001
NCCN low-risk group	1.326	0.451–3.896	0.608
NCCN intermediate-risk group	0.432	0.246–0.761	0.004
NCCN high-risk group	0.634	0.449–0.895	0.010
NCCN very-high-risk group	0.432	0.234–0.797	0.007

HR, Hazard ratio; CI, Confidence Interval; NCCN, National Comprehensive Cancer Network; BCFFS, Biochemical failure-free survival; BCF, Biochemical failure.

## Data Availability

Data sharing is not applicable to this article.

## Supplementary Materials

The following are available online at https://www.mdpi.com/article/10.3390/cancers13112732/s1, Figure S1: Distribution of total dose in biologically effective dose (BED) against different fractional doses (a) or different radiotherapy (RT) modalities (b), Figure S2: Temporal trends according to treatment year on specific radiotherapy (RT) techniques: (a) patients with intensity-modulated RT (IMRT) or proton therapy vs. 3-dimensional conformal RT, (b) patients with hypofractionation (HF) or ultra-HF vs. conventional fractionation (CF) RT, (c) patients with higher RT dose (≥179 Gy_1.5_) vs. lower RT dose (<179 Gy_1.5_), and (d) patients with combined RT-related factors (IMRT, HF, and higher dose (≥179 Gy_1.5_)), Table S1: Prostate cancer risk stratification criteria for different risk-grouping systems, Table S2: Summary of treatment outcomes, Table S3: Toxicity rates stratified by RTOG radiation toxicity criteria in all patients (*n* = 1573), Table S4: Toxicity rates stratified by RTOG radiation toxicity criteria in patients treated with a combination of three factors (intensity-modulated radiotherapy (IMRT), hypofractionation, and higher dose (≥ 179 Gy_1.5_)) (*n* = 829).

## Appendix A

Based on the National Comprehensive Cancer Network (NCCN) guidelines, the 5- and 10-year biochemical failure-free survival rates were 91.6% and 75.4% in the low-risk group, 83.7% and 65.3% in the intermediate-risk group, 80.6% and 54.7% in the high-risk group, and 77.1% and 51.9% in the very-high-risk group, respectively. The actual biochemical failure rates were 8.5%, 15.4%, 22.1%, and 25.6% in the low-, intermediate-, high-, and very-high-risk groups, respectively. The 5- and 10-year overall survival rates were 91.6% and 75.4% in the low-risk group, 83.7% and 65.3% in the intermediate-risk group, 80.6% and 54.7% in the high-risk group, and 77.1% and 51.9% in the very-high-risk group, respectively. The 5- and 10-year cause-specific survival rates were all 100% in the low-risk group, 99.7% and 98.8% in the intermediate-risk group, 98.7% and 95.3% in the high-risk group, and 96.4% and 93.6% in the very-high-risk group, respectively.
