# Supplementary material for: Optimizing External Beam Radiotherapy as per the Risk Group of Localized Prostate Cancer: A Nationwide Multi-Institutional Study (KROG 18-15)
Source: Cancers (Basel). 2021 May 31;13(11):2732. doi: 10.3390/cancers13112732 (PMC8198120; doi:10.3390/cancers13112732)
Supplement: Supplementary file 1 [file cancers-13-02732-s001.zip › cancers-1186594-supplementary.pdf]

Supplementary Figure

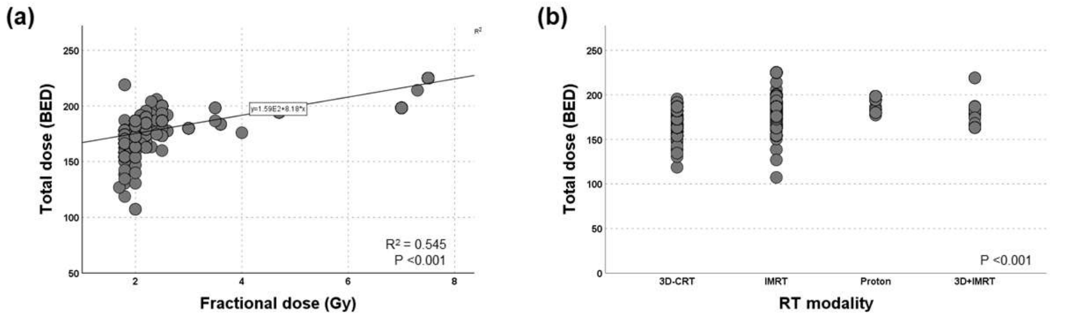

	Fractional dose			
	1.8-2.0 Gy/fr	2.1-2.6 Gy/fr	3.0-4.7 Gy/fr	≥7 Gy/fr
Total BED (mean, Gy)	167.79	185.45	187.29	214.85

	RT modality			
	3D-CRT	IMRT	Proton	3D+IMRT
Total BED (mean, Gy)	166.00	185.08	191.33	177.71

Figure S1. Distribution of total dose in biologically effective dose (BED) against different fractional doses (a) or different radiotherapy (RT) modalities (b). Total irradiated dose increased significantly as the fractional dose increased or with the use of modern RT modalities such as intensity-modulated RT (IMRT) or proton therapy (all $p<0.001$).

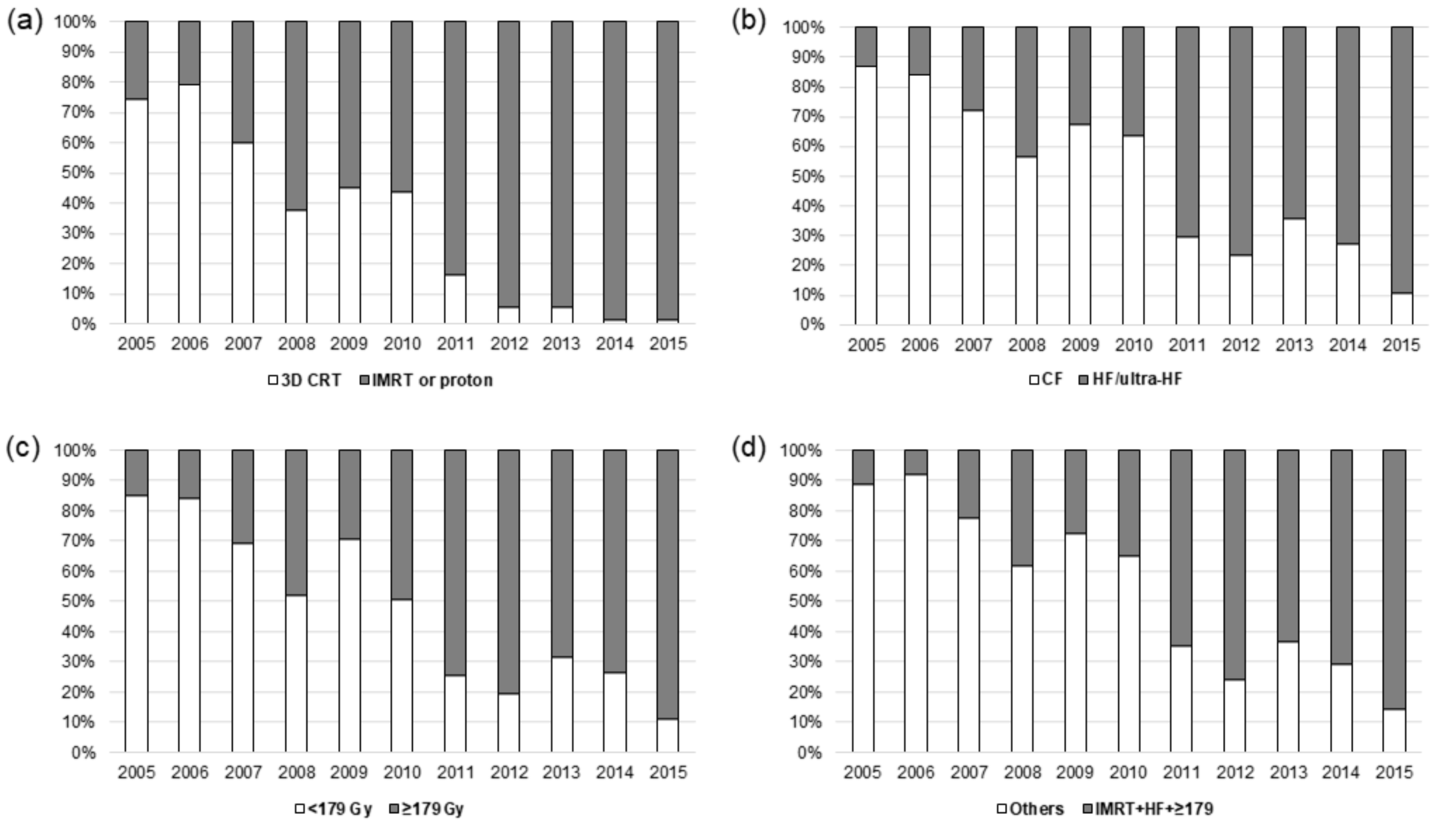

Figure S2. Temporal trends according to treatment year on specific radiotherapy (RT) techniques: (a) patients with intensity-modulated RT (IMRT) or proton therapy vs. 3 dimensional conformal RT, (b) patients with hypofractionation (HF) or ultra-HF vs. conventional fractionation (CF) RT, (c) patients with higher RT dose [≥179 $Gy_{1.5}$] vs. lower RT dose [<179 $Gy_{1.5}$], and (d) patients with combined RT-related factors (IMRT, HF, and higher dose [≥179 $Gy_{1.5}$]).

SUPPLEMENTARY TABLES

Table S1. Prostate cancer risk stratification criteria for different risk grouping systems.

	**Very low risk**	**Low risk**	**Intermediate risk**		**High risk**	**Very high risk**
			**Favorable**	**Unfavorable**		
**D'Amico**		PSA ≤10 and<br>GS ≤6 and<br>cT1c-T2a	PSA >10–20 or<br>GS 7 or<br>cT2b		PSA >20 or<br>GS 8–10 or<br>cT2c	
**AUA**		PSA <10 and<br>GS ≤6 (ISUP 1) and<br>cT1c-T2a	PSA 10–20 or<br>GS 7 (ISUP 2–3) or<br>cT2b		PSA >20 or<br>GS >7 (ISUP 4–5) or<br>cT2c	
**CPG**		PSA <10 and<br>GS 6 (ISUP 1) and<br>cT1-T2	PSA 10–20 or<br>GS 3 + 4 (ISUP 2)<br>and<br>cT1-T2	PSA 10–20 and<br>GS 3 + 4 (ISUP 2) and<br>cT1-T2<br>or<br>GS 4 + 3 (ISUP 3)<br>and<br>cT1-T2	PSA >20 or<br>GS 8 (ISUP 4) or<br>cT3	More than one of PSA>20, GS 8 (ISUP 4), cT3 or<br>GS 9–10 (ISUP 5) or<br>cT4
**NCCN**	PSA <10 and<br>GS ≤6 (ISUP 1)<br>and<br>cT1c and<br><3 positive cores<br>and<br>≤50% cancer in<br>each core and<br>PSA density<br><0.15	PSA <10 and<br>GS ≤6 (ISUP 1) and<br>cT1-T2a	PSA 10–20 or<br>GS 3 + 4 (ISUP 2) or<br>cT2b-T2c and<br><50% positive cores	PSA 10–20 or<br>GS 3 + 4/4 + 3<br>(ISUP 2–3) or<br>cT2b-T2c	PSA >20 or<br>GS 4 + 4/4 + 5 (ISUP 4–5)<br>or<br>cT3a	cT3b-T4 or<br>Primary Gleason pattern 5 or<br>>4 cores with grade group 4 or 5

AUA, American Urological Association; CPG, Cambridge Prognostic Groups; NCCN, National Comprehensive Cancer Network; PSA, Prostate-Specific Antigen; GS, Gleason score; ISUP, International Society of Urological Pathology

Table S2. Summary of treatment outcomes.

Rate	No.	%
Overall death	148	9.4
Disease-related death	33	2.1
Any recurrence	319	20.3
Both - BCF first (or concurrently)	96	6.1
Both - CF first	19	1.2
BCF only	189	12.0
CF only	15	1.0
Overall BCF events	304	19.3
Timing (after first diagnosis, months)	Median 46 (2–211)	
Overall CF events	131	8.3
Local	42	2.7
Timing (after first diagnosis, days)	Median 531 (2–2524)	
Regional	28	1.8
Timing (after first diagnosis, days)	Median 591 (4–2735)	
Distant	90	5.7
Timing (after first diagnosis, days)	Median 413 (1–3380)	

BCF, Biochemical failure; CF, Clinical failure

Table S3. Clinical and treatment characteristics of patients after performing propensity score matching (matched group: IMRT/proton (n = 399) and 3D CRT (n = 399)).

		Before matching				P value	After matching				P value
		IMRT/Proton (n =1174)		3D-CRT (n =399)			IMRT/Proton (n =399)		3D-CRT (n =399)		
		No.	%	No.	%		No.	%	No.	%	
**Age (year, mean)**		71.72		70.89		0.028	71.04		70.89		0.774
**T stage**	**T2, 3, 4**	1030	87.7	325	81.5	0.004	326	81.7	325	81.5	0.927
	**T1**	144	12.3	74	18.5		73	18.3	74	18.5	
**Gleason score**	**<9**	1000	85.2	332	83.2	0.345	350	87.7	332	83.2	0.071
	**≥9**	174	14.8	67	16.8		49	12.3	67	16.8	
**iPSA**	**<12**	596	50.8	186	46.6	0.152	193	48.4	186	46.6	0.620
	**≥12**	578	49.2	213	53.4		206	51.6	213	53.4	
**NCCN risk group**	**Low**	134	11.4	43	10.8	0.279	65	16.3	43	10.8	0.002
	**Intermediate**	304	25.9	104	26.1		120	30.1	104	26.1	
	**High**	578	49.2	183	45.9		176	44.1	183	45.9	
	**Very high**	158	13.5	69	17.3		38	9.5	69	17.3	
**RT volume**	**Prostate**	762	64.9	266	66.7	0.140	329	82.5	266	66.7	<0.001
	**Whole pelvis**	401	34.2	133	33.3		65	16.3	133	33.3	
	**Unknown**	11	0.9	0	0.0		5	1.3	0	0.0	
**RT scheme**	**CF**	457	38.9	378	94.7	<0.001	378	94.7	378	94.7	0.999
	**HF**	717	61.1	21	5.3		21	5.3	21	5.3	
**Total dose, BED ($Gy_{1.5}$) (mean)**		185.08		166.61		<0.001	186.24		166.61		<0.001
**ADT**	**No**	426	36.3	163	40.9	0.104	177	44.4	163	40.9	0.316
	**Yes**	748	63.7	236	59.1		222	55.6	236	59.1	

3D CRT, 3-dimensional conformal radiotherapy; IMRT, Intensity-modulated radiotherapy; PSA, Prostate-Specific Antigen; NCCN, National Comprehensive Cancer Network; RT, Radiotherapy; SV, Seminal vesicle; CF, conventional fractionation; HF, hypofractionation; BED, Biologically effective dose; ADT, Androgen deprivation therapy

Table S4. Results of univariate and multivariate analyses for biochemical failure-free survival in all patients and each NCCN risk group after performing propensity score matching.

**All patients**	**Univariate**		**Multivariate**	
	**p value**	**HR (95% CI)**	**p value**	**HR (95% CI)**
T stage (T1 vs. T2, T3, T4)	0.022	0.688 (0.499-0.948)	0.084	0.740 (0.526-1.042)
Gleason score (≥9 vs. <9)	0.002	1.600 (1.188-2.155)	<0.001	1.833 (1.328-2.528)
initial PSA (≥12 vs. <12)	0.006	1.403 (1.104-1.783)	0.003	1.499 (1.148-1.958)
RT volume (Pelvis vs. Prostate±SV)	0.699	1.027 (0.896-1.178)	0.673	1.062 (0.804-1.403)
RT scheme (CF vs. HF)	0.526	1.110 (0.804-1.534)	0.353	0.778 (0.458-1.321)
BED ≥179 Gy1.5 vs. <179 Gy1.5	0.153	0.816 (0.617-1.079)	0.090	0.687 (0.445-1.061)
RT modality (3D vs. IMRT/proton)	0.396	1.111 (0.871-1.416)	0.742	1.046 (0.800-1.369)
ADT combination (Yes vs. No)	0.031	0.741 (0.584-0.939)	<0.001	0.539 (0.412-0.707)

**Low risk group patients**	**Univariate**		**Multivariate**	
	**p value**	**HR (95% CI)**	**p value**	**HR (95% CI)**
T stage (T1 vs. T2, T3, T4)	0.506	1.383 (0.533-3.590)	0.462	1.470 (0.527-4.106)
RT volume (Pelvis vs. Prostate±SV)	0.541	1.578 (0.366-6.798)	0.422	1.891 (0.400-8.946)
RT scheme (CF vs. HF)	0.404	0.676 (0.269-1.696)	0.454	0.525 (0.097-2.833)
BED ≥179 Gy1.5 vs. <179 Gy1.5	0.693	1.196 (0.492-2.908)	0.877	0.888 (0.198-3.981)
RT modality (3D vs. IMRT/proton)	0.976	1.014 (0.417-2.465)	0.733	1.253 (0.344-4.563)
ADT combination (Yes vs. No)	0.402	0.627 (0.211-1.868)	0.578	0.724 (0.233-2.256)

**Intermediate risk patients**	**Univariate**		**Multivariate**	
	**p value**	**HR (95% CI)**	**p value**	**HR (95% CI)**
T stage (T1 vs. T2, T3, T4)	0.786	0.932 (0.562-1.547)	0.940	0.980 (0.585-1.644)
initial PSA (≥12 vs. <12)	0.803	0.941 (0.581-1.524)	0.845	0.952 (0.580-1.563)
RT volume (Pelvis vs. Prostate±SV)	0.709	0.869 (0.416-1.814)	0.495	0.770 (0.364-1.631)
RT scheme (CF vs. HF)	0.609	1.170 (0.641-2.137)	0.639	1.357 (0.379-4.862)
BED ≥179 Gy1.5 vs. <179 Gy1.5	0.700	0.896 (0.514-1.563)	0.927	0.948 (0.304-2.959)
RT modality (3D vs. IMRT/proton)	0.811	1.059 (0.663-1.691)	0.730	0.908 (0.526-1.568)
ADT combination (Yes vs. No)	0.044	0.602 (0.367-0.987)	0.035	0.572 (0.340-0.962)

**High risk patients**	**Univariate**		**Multivariate**	
	**p value**	**HR (95% CI)**	**p value**	**HR (95% CI)**
T stage (T1 vs. T2, T3, T4)	0.996	0.998 (0.505-1.972)	0.973	1.021 (0.504-2.034)
Gleason score (≥9 vs. <9)	0.871	0.956 (0.5591.638)	0.271	1.368 (0.783-2.390)
initial PSA (≥12 vs. <12)	0.917	1.022 (0.682-1.530)	0.358	1.222 (0.797-1.876)
RT volume (Pelvis vs. Prostate±SV)	0.781	1.053 (0.731-1.518)	0.484	0.873 (0.595-1.279)
RT scheme (CF vs. HF)	0.988	0.996 (0.606-1.639)	0.186	0.574 (0.252-1.306)
BED ≥179 Gy1.5 vs. <179 Gy1.5	0.041	0.727 (0.476-0.956)	0.029	0.468 (0.237-0.924)
RT modality (3D vs. IMRT/proton)	0.099	1.349 (0.946-1.925)	0.071	1.420 (0.970-2.078)
ADT combination (Yes vs. No)	<0.001	0.442 (0.308-0.633)	<0.001	0.391 (0.265-0.577)

**Very-high risk patients**	**Univariate**		**Multivariate**	
	**p value**	**HR (95% CI)**	**p value**	**HR (95% CI)**
Gleason score (≥9 vs. <9)	0.009	2.658 (1.281-5.515)	0.021	2.531 (1.147-5.582)
initial PSA (≥12 vs. <12)	0.068	1.811 (0.957-3.427)	0.161	1.670 (0.815-3.420)
RT volume (Pelvis vs. Prostate±SV)	0.554	1.190 (0.669-2.115)	0.319	1.364 (0.741-2.510)
RT scheme (CF vs. HF)	0.656	0.792 (0.283-2.216)	0.708	0.760 (0.182-3.183)
BED ≥179 Gy1.5 vs. <179 Gy1.5	0.892	1.046 (0.544-2.013)	0.876	1.066 (0.477-2.381)
RT modality (3D vs. IMRT/proton)	0.035	0.537 (0.301-0.958)	0.019	0.467 (0.248-0.880)
ADT combination (Yes vs. No)	0.513	0.792 (0.394-1.594)	0.077	0.481 (0.213-1.084)

HR, Hazard ratio; CI, Confidence Interval; PSA, Prostate-Specific Antigen; RT, Radiotherapy; SV, Seminal vesicle; 3D, 3-dimensional conformal radiotherapy; IMRT, Intensity-modulated radiotherapy; CF, conventional fractionation; HF, hypofractionation; BED, Biologically effective dose; ADT, Androgen deprivation therapy

Table S5. Toxicity rates stratified by RTOG radiation toxicity criteria in all patients (n = 1,573).

Toxicity	Grade	No.	%
Acute GU toxicity	No	983	62.5
	Grade 1	421	26.8
	Grade 2	161	10.2
	Grade 3	8	0.5
Acute GI toxicity	No	1302	82.8
	Grade 1	173	11.0
	Grade 2	97	6.2
	Grade 3	1	0.1
Late GU toxicity	No	1278	81.2
	Grade 1	85	5.4
	Grade 2	195	12.4
	Grade 3	15	1.0
Late GI toxicity	No	1340	85.2
	Grade 1	131	8.3
	Grade 2	79	5.0
	Grade 3	23	1.5

GU, Genitourinary; GI, Gastrointestinal

*** Toxicity rates according to follow-up duration:**

		**<5 years follow-up**		**≥5 years of follow-up**		
		**No.**	**%**	**No.**	**%**	**p value**
Acute GU toxicity	No	285	29.0	698	71.0	0.303
	Grade 1	140	33.3	281	66.7	
	Grade 2	43	26.7	118	73.3	
	Grade 3	3	37.5	5	62.5	
Acute GI toxicity	No	403	31.0	899	69.0	0.214
	Grade 1	41	23.7	132	76.3	
	Grade 2	27	27.8	70	72.2	
	Grade 3	0	0.0	1	100.0	
Late GU toxicity	No	386	30.2	892	69.8	0.833
	Grade 1	24	28.2	61	71.8	
	Grade 2	58	29.7	137	70.3	
	Grade 3	3	20.0	12	80.0	
Late GI toxicity	No	396	29.6	944	70.4	0.208
	Grade 1	43	32.8	88	67.2	
	Grade 2	21	26.6	58	73.4	
	Grade 3	11	47.8	12	52.2	

GU, Genitourinary; GI, Gastrointestinal

		**<5 years follow-up**		**≥5 years of follow-up**		
		No.	%	No.	%	p value
Acute GU toxicity	<Grade 2	425	30.3	979	69.7	0.413
	≥Grade 2	46	27.2	123	72.8	
Acute GI toxicity	<Grade 2	444	30.1	1031	69.9	0.593

	≥Grade 2	27	27.6	71	72.4	
Late GU toxicity	<Grade 2	410	30.1	953	69.9	0.761
	≥Grade 2	61	29.0	149	71.0	
Late GI toxicity	<Grade 2	439	29.8	1032	70.2	0.744
	≥Grade 2	32	31.4	70	68.6	

GU, Genitourinary; GI, Gastrointestinal

*** Mean values of follow-up duration between patients who underwent toxicities and the rest**

		**Follow-up duration (mean)**	**p value**
**Acute GU toxicity**	**No**	81.24	0.323
	**Yes**	79.57	
**Acute GI toxicity**	**No**	79.75	0.021
	**Yes**	84.74	
**Late GU toxicity**	**No**	80.72	0.791
	**Yes**	80.16	
**Late GI toxicity**	**No**	80.62	0.039
	**Yes**	80.58	

GU, Genitourinary; GI, Gastrointestinal

		**Follow-up duration (mean)**	**p value**
**Acute GU toxicity**	**<Grade 2**	80.57	0.893
	**≥Grade 2**	80.93	
**Acute GI toxicity**	**<Grade 2**	80.52	0.654
	**≥Grade 2**	82.03	
**Late GU toxicity**	**<Grade 2**	80.94	0.261
	**≥Grade 2**	78.50	
**Late GI toxicity**	**<Grade 2**	80.68	0.740
	**≥Grade 2**	79.59	

GU, Genitourinary; GI, Gastrointestinal

Table S6. Toxicity rates stratified by RTOG radiation toxicity criteria in patients treated with a combination of three factors (intensity-modulated radiotherapy (IMRT), hypofractionation, and higher dose (≥ 179 $Gy_{1.5}$)) (n = 829).

Toxicity	Grade	No.	%
Acute GU toxicity	No	496	59.8
	Grade 1	236	28.5
	Grade 2	89	10.7
	Grade 3	8	1.0
Acute GI toxicity	No	697	84.1
	Grade 1	77	9.3
	Grade 2	55	6.6
	Grade 3	0	0.0
Late GU toxicity	No	630	76.0
	Grade 1	53	6.4
	Grade 2	138	16.6
	Grade 3	8	1.0
Late GI toxicity	No	695	83.8
	Grade 1	75	9.0
	Grade 2	46	5.5
	Grade 3	13	1.6

GU, Genitourinary; GI, Gastrointestinal

*** Toxicity rates according to follow-up duration:**

		<5 years follow-up		≥5 years of follow-up		
		**No.**	**%**	**No.**	**%**	**p value**
Acute GU toxicity	No	124	41.5	175	58.5	0.528
	Grade 1	73	45.9	86	54.1	
	Grade 2	28	36.4	49	63.6	
	Grade 3	2	33.3	4	66.7	
Acute GI toxicity	No	192	43.3	251	56.7	0.026
	Grade 1	14	25.5	41	74.5	
	Grade 2	21	48.8	22	51.2	
	Grade 3	0	0.0	0	0.0	
Late GU toxicity	No	166	43.9	212	56.1	0.833
	Grade 1	13	32.5	27	67.5	
	Grade 2	45	39.1	70	60.9	
	Grade 3	3	37.5	5	62.5	
Late GI toxicity	No	188	40.4	277	59.6	0.208
	Grade 1	19	45.2	23	54.8	
	Grade 2	13	54.2	11	45.8	
	Grade 3	7	70.0	3	30.0	

GU, Genitourinary; GI, Gastrointestinal

		<5 years follow-up		≥5 years of follow-up		
		No.	%	No.	%	p value
Acute GU toxicity	<Grade 2	197	43.0	261	57.0	0.243
	≥Grade 2	30	36.1	53	63.9	
Acute GI toxicity	<Grade 2	206	41.4	292	58.6	0.341
	≥Grade 2	21	48.8	22	51.2	
Late GU toxicity	<Grade 2	179	42.8	239	57.2	0.453
	≥Grade 2	48	39.0	75	61.0	
Late GI toxicity	<Grade 2	207	40.8	300	59.2	0.04
	≥Grade 2	20	58.8	14	41.2	

GU, Genitourinary; GI, Gastrointestinal
